# Perspectives on HIV pre-exposure prophylaxis (PrEP) utilization and related intervention needs among people who inject drugs

**DOI:** 10.1186/s12954-018-0263-5

**Published:** 2018-11-12

**Authors:** K. B. Biello, A. R. Bazzi, M. J. Mimiaga, D. L. Biancarelli, A. Edeza, P. Salhaney, E. Childs, M. L. Drainoni

**Affiliations:** 1 Departments of Behavioral and Social Sciences and Epidemiology, Center for Health Equity Research, Brown University School of Public Health, Box G-S121-8, Providence, RI 02912 USA; 2 Center for Health Equity Research, Brown University, Providence, RI USA; 3 The Fenway Institute, Fenway Health, Boston, MA USA; 4 Department of Community Health Sciences, Boston University School of Public Health, Boston, MA USA; 5 Department of Psychiatry and Human Behavior, Brown University Alpert Medical School, Providence, RI USA; 6 Department of Health Law, Policy & Management, Boston University School of Public Health, Boston, MA USA; 7 Department of Behavioral and Social Sciences, Brown University School of Public Health, Providence, RI USA; 8 Section of Infectious Diseases, Department of Medicine, Boston University School of Medicine, Boston, MA USA; 9 Evans Center for Implementation and Improvement Sciences, Boston University School of Medicine, Boston, MA USA; 10 Center for Healthcare Organization and Implementation Research, Edith Nourse Rogers Memorial Veterans Hospital, Bedford, MA USA

**Keywords:** Pre-exposure prophylaxis, HIV prevention, PWID, Intervention development

## Abstract

**Background:**

Antiretroviral pre-exposure prophylaxis (PrEP) is clinically efficacious and recommended for HIV prevention among people who inject drugs (PWID), but uptake remains low and intervention needs are understudied. To inform the development of PrEP interventions for PWID, we conducted a qualitative study in the Northeastern USA, a region where recent clusters of new HIV infections have been attributed to injection drug use.

**Methods:**

We conducted qualitative interviews with 33 HIV-uninfected PWID (hereafter, “participants”) and 12 clinical and social service providers (professional “key informants”) in Boston, MA, and Providence, RI, in 2017. Trained interviewers used semi-structured interviews to explore PrEP acceptability and perceived barriers to use. Thematic analysis of coded data identified multilevel barriers to PrEP use among PWID and related intervention strategies.

**Results:**

Among PWID participants (*n* = 33, 55% male), interest in PrEP was high, but both participants and professional key informants (*n* = 12) described barriers to PrEP utilization that occurred at one or more socioecological levels. Individual-level barriers included low PrEP knowledge and limited HIV risk perception, concerns about PrEP side effects, and competing health priorities and needs due to drug use and dependence. Interpersonal-level barriers included negative experiences with healthcare providers and HIV-related stigma within social networks. Clinical barriers included poor infrastructure and capacity for PrEP delivery to PWID, and structural barriers related to homelessness, criminal justice system involvement, and lack of money or identification to get prescriptions. Participants and key informants provided some suggestions for strategies to address these multilevel barriers and better facilitate PrEP delivery to PWID.

**Conclusions:**

In addition to some of the facilitators of PrEP use identified by participants and key informants, we drew on our key findings and behavioral change theory to propose additional intervention targets. In particular, to help address the multilevel barriers to PrEP uptake and adherence, we discuss ways that interventions could target information, self-regulation and self-efficacy, social support, and environmental change. PrEP is clinically efficacious and has been recommended for PWID; thus, development and testing of strategies to improve PrEP delivery to this high-risk and socially marginalized population are needed.

## Introduction

People who inject drugs (PWID) are disproportionately affected by HIV/AIDS [1], accounting for 7–10% of new HIV infections in the USA annually [1, 2]. Needle syringe programs (NSPs) are effective in reducing HIV transmission via injection drug use and provide other essential harm reduction and health services to PWID [3]. However, even with NSP coverage expanding, NSP access is insufficient in some areas [4], and national behavioral surveillance reveals persistent risk behavior engagement in PWID populations. For example, the 2015 National HIV Behavioral Surveillance survey in 20 US cities found that only half (52%) of HIV-uninfected PWID utilized NSPs and even fewer (34%) consistently used sterile syringes in the past year [2]. Furthermore, HIV-related risk behaviors are common in this population, with 72% reporting past year receptive syringe sharing or condomless sex [2]. As evidenced by recent HIV outbreaks linked to injection drug use [5], the introduction of HIV into networks of PWID with frequent syringe sharing is an important concern. Furthermore, transmission of hepatitis C virus (HCV), considered a harbinger of HIV outbreaks [6], has significantly increased over the past decade [7]. Combined with these emerging behavioral and epidemiologic trends [8], the increasing prevalence of opioid use and injection across the USA [9, 10] suggests that improved access to HIV and other prevention services for PWID are urgently needed [11].

Antiretroviral pre-exposure prophylaxis (PrEP) is CDC recommended for HIV prevention among high-risk PWID [12]. In the only clinical trial to test PrEP among PWID to date, the Bangkok Tenofovir Study [13], adherence to daily PrEP was lower among men, younger participants (≤ 40 yrs), those with recent incarceration, and methamphetamine users [14]. However, while this trial demonstrated the clinical efficacy of PrEP in preventing HIV acquisition among PWID in Thailand, the feasibility of and cost-effectiveness of PrEP delivery to PWID in diverse contexts have been questioned [15–19]. Furthermore, very little is known about the acceptability or accessibility of PrEP for HIV prevention among PWID in North America [20, 21], where PrEP is recommended for PWID but actual uptake in this population has been low, and knowledge, acceptability, and potential adherence challenges remain poorly understood [22–26]. Although some recent research has explored PrEP interest and delivery for patients engaged in methadone for treatment of opioid use disorder [27–29], research with individuals actively injecting drugs is needed [30]. To understand barriers to PrEP uptake and inform the development of interventions to improve PrEP utilization among PWID at risk for HIV acquisition, we conducted a qualitative study in the Northeastern USA, where injection of opioids and other drugs is increasingly widespread [9, 31] and has been linked to HCV transmission and recent clusters of new HIV infections attributed to injection drug use [32]. In particular, the increasing prevalence of fentanyl exposure among PWID in Massachusetts and Rhode Island has raised concerns about increased frequency of injection and other risk behaviors for HIV transmission [32, 33].

## Methods

### Study design and sample

As previously described [30], to recruit high-risk PWID, we partnered with community-based organizations (CBOs) experienced in conducting outreach and service delivery to this population including NSPs and drop-in HIV/HCV testing centers in Boston, MA, and Providence, RI. Although these organizations did not directly provide PrEP-related services at the time of the study, they provided a range of other HIV prevention and harm reduction services as well as supported referrals to healthcare, drug treatment, and social services. To help recruit PWID, CBO staff, with whom we regularly communicated about our sampling strategy, first privately approached known PWID to explain the study. Research study staff then screened interested individuals for eligibility, which included being ≥ 18 years of age, being HIV-uninfected (self-report), and injecting any drugs in the past month. Eligible PWID (hereafter, “participants”) provided verbal informed consent, which was documented by study staff. In line with purposive sampling methods [34, 35], we continually monitored enrollment characteristics and regularly communicated with CBO staff to adjust our recruitment strategies as needed to obtain a demographically diverse, high-risk sample (i.e., oversampling women and racial/ethnic minorities and those reporting recent [past month] receptive syringe sharing and condomless sex). To understand provider perspectives, we worked with our professional networks to recruit individuals ≥ 18 years of age with professional experience providing PrEP or other health or harm reduction services to PWID (either through clinical practices or CBOs). These “key informants” were not current PWID themselves but could provide unique professional perspectives on PrEP delivery to the local PWID population. Eligible key informants provided verbal informed consent, also documented by study staff. The institutional review board of Boston University Medical Center approved all study protocols.

### Data collection

From October 2016 to October 2017, trained qualitative interviewers conducted confidential interviews in private offices or other spaces within CBOs. Interviewers administered a brief demographic assessment, including the following question on PrEP knowledge:

A method now being used to prevent HIV is called pre-exposure prophylaxis, or PrEP. PrEP is a way for people who don’t have HIV but who are at risk of getting it (through sex or injection drugs) to prevent HIV infection by taking a pill every day. Sometimes this is called Truvada or the ‘HIV prevention pill.’ Before today, had you heard about PrEP, Truvada or the ‘HIV prevention pill’?

Interviewers then used field-tested semi-structured interview guides with open-ended questions to explore risk behaviors, HIV prevention and health service utilization, and PrEP acceptability and perceptions regarding various aspects of uptake and adherence. If a participant did not know what PrEP was, the following description was read:

PrEP is basically an antiretroviral pill that can be taken every day by people who don’t have HIV yet but who are at risk of getting it (through sex or injecting drugs). Studies have shown that it is very effective in preventing HIV if taken as prescribed.

Other example questions included, “Someone who is interested in PrEP needs to meet with a healthcare provider to discuss PrEP and their risk for HIV... [and have] testing for HIV…What would it be like for you to do this? Why?” and PrEP must be taken every day around the same time of day, for a period of time. What would it be like for you to do this? Why?

Detailed probes explored specific challenges and potential facilitators of PrEP utilization (e.g., relating to service delivery locations, types of providers, communicating with providers, adherence difficulties and helpful reminder systems, medication storage, and coping with side effects). Key informants also completed brief demographic quantitative assessments and qualitative interviews using field-test semi-structured guides with open-ended questions about experience working in HIV or other service provision, perspectives on PrEP as HIV prevention for PWID and potential barriers. Examples of question include, “Tell me about your job and related experience working with people who inject drugs?” and “What do you think is preventing [PrEP] delivery to this population?” and “What are possible solutions?”

Interviews lasted ~ 45 min with participants and ~ 30 min with key informants. Interviews were audio-recorded and professionally transcribed for text analysis. We continued recruiting and interviewing participants until determining through regular team discussions that we had reached thematic saturation, or the point after which collecting additional data would be unlikely to yield substantially new or different insights on key topics of interest [35, 36].

### Data analysis

We reviewed transcripts for quality and to identify emergent themes [37] and employed a collaborative codebook development process [38, 39]. First, six research team members (including three investigators and three trained qualitative research assistants) independently read three selected transcript excerpts to generate potential codes and definitions based on topics of interest (i.e., key domains and questions from interview guides and topics that emerged during interviews and were discussed in regular team meetings). We discussed and compiled potential codes into a preliminary codebook that team members then independently applied to a set of three full transcripts. We compared code application, discussed discrepancies, and modified the codebook for application to another set of transcripts. Through two additional rounds of this process, we continued refining codes and definitions until reaching consensus on the final codebook. Three analysts then used NVivo (v11) to independently apply final codes to their assigned transcripts. Coding was supervised and monitored by a lead analyst who held regular discussions of coding progress through weekly calls. In-depth, thematic analysis then involved using a primarily deductive approach to synthesize data coded for perceived barriers to PrEP use and suggestions for intervention strategies in order to identify and clarify key themes and connections between themes. Findings are illustrated in the sections below using representative quotes with pseudonyms to protect confidentiality.

## Results

### Study sample characteristics

Among 33 participants (*n* = 16 in Boston, *n* = 17 in Providence), median age was 36 years (range 24–62), 18 (55%) identified as male, and 20 (61%) injected at least daily (Table 1). Common drugs injected were heroin (94%), cocaine (70%), crack (39%), and methamphetamine (33%). Nearly two thirds reported any past month receptive or distributive syringe sharing (i.e., receiving or giving used syringes to/from others; 64%). Among those with ≥ 1 sex partner in the past 3 months (*n* = 27), over half reported condomless sex (59%). Key informants (*n* = 8 in Boston, *n* = 4 in Providence) worked as clinical and CBO-based social service providers directly interfacing with PWID (Table 2). Overall, in their various roles as PrEP and HIV treatment providers, primary care clinicians, infectious disease and addiction medicine specialists, and CBO program managers and outreach staff, more than half of the key informants had worked with PWID for 11+ years.

**Table 1:** Characteristics of people who inject drugs in study sample (*n* = 33)

	*n* (%)*
Socio-demographics	
City	
Boston	16 (48)
Providence	17 (52)
Age in years; median (interquartile range; IQR)	36 (32–48)
Race (categories are not mutually exclusive)	
American Indian or Alaska Native	3 (9)
Black or African American	7 (21)
White	22 (67)
Other	5 (15)
Ethnicity: Hispanic/Latino	8 (24)
Gender	
Male	18 (55)
Female	13 (39)
Transgender	1 (3)
Genderqueer	1 (3)
Sexual orientation	
Heterosexual or “Straight”	21 (64)
Bisexual	8 (24)
Homosexual or gay	4 (12)
Educational attainment	
Less than high school	9 (27)
High school or GED	13 (39)
Some college	11 (33)
Employment status: unemployed	23 (70)
Health insurance: has public health insurance	32 (97)
Sexual health and behaviors	
HIV testing, past year (*n* = 32)	30 (93)
STI testing, past year	24 (73)
Diagnosed with HCV, ever	26 (79)
Time since HCV diagnosis in years; median (interquartile range; IQR)	5 (1.5–9)
Number of sex partners, past 3 months	
0	6 (18)
1	12 (36)
2+	17 (45)
Condom use, past 3 months (vaginal or anal sex; *n* = 27 with ≥ 1 sex partner)	
Never/rarely	12 (45)
Sometimes/usually	10 (37)
Always	5 (19)
Substance use behaviors	
Frequency of drug injection, past 3 months	
Less than once a month	2 (6)
1 to 3 days a month	2 (6)
Once a week	1 (3)
2 to 6 days a week	8 (24)
Once a day everyday	3 (9)
2+ times a day everyday	17 (51)
Drugs injected, past 3 months (not mutually exclusive)	
Heroin	31 (94)
Prescription opioids	3 (9)
Methadone	1 (3)
Cocaine	23 (70)
Crack	13 (39)
Crystal methamphetamine	11 (33)
Cocaine/heroin combination (“Speedball”)	12 (36)
Current syringe access (not mutually exclusive)	
Needle syringe program (NSP)	27 (82)
Pharmacy	10 (30)
Other people	5 (15)
Any distributive syringe sharing, past month (*n* = 32)	15 (47)
Any receptive syringe sharing, past month	19 (57)
Any distributive or receptive syringe sharing, past month	21 (64)
Any shared injection paraphernalia (cookers, cottons, rinse water), past month	21 (64)
PrEP knowledge and experience	
Had heard of PrEP prior to study	12 (36)
Had taken PrEP prior to study	1 (3)
Perceived change in risk behaviors if taking PrEP in future (*n* = 32)	
Yes: increase	5 (16)
Yes: decrease	7 (22)
No change	20 (63)
Likelihood of using PrEP in future	
Extremely unlikely	0 (0)
Unlikely	3 (9)
Undecided	13 (39)
Likely	10 (30)
Extremely likely	7 (21)

*May exceed 100% when categories were not mutually exclusive

**Table 2:** Employment characteristics for key informants (*n* = 12)

	*N*
Organization type*	
Drop-in HIV/STI/HCV testing center	7
HIV primary care clinic/hospital	5
Methadone clinic	1
Substance use clinic	1
Needle syringe program (NSP)	3
State public health department	1
Job titles	
Clinician/researcher	5
Program coordinator/manager	5
Outreach worker/navigator	2
Years of experience in HIV and/or PWID	
0–5	3
6–10	3
11+	6

*May exceed 100% when categories were not mutually exclusive

Although interest in PrEP for HIV prevention in PWID was high [30], participants and key informants described numerous barriers to PrEP uptake and adherence that occurred at one or more socioecological levels. These individual, interpersonal, clinical, and structural level barriers often interacted to further complicate potential PrEP utilization. Barriers and some suggested strategies for addressing them are described in the sections below.

### Individual-level barriers

Individual-level barriers to PrEP utilization included low PrEP knowledge and limited HIV risk perception, concerns about PrEP side effects, and competing health priorities and needs due to drug use and dependence. First, as detailed elsewhere [30], knowledge of PrEP, including how it works and its advantages and disadvantages, was extremely low among participants. Most participants had never heard of PrEP or had vague familiarity with it, sometimes associating it with gay men which key informants described as a potential barrier to PrEP use among PWID who did not identify as gay or perceive themselves to be at high risk for HIV acquisition. As one physician in Boston reported, “I think a lot of our MSM patients are more familiar with PrEP because it might be more talked about and known. But our patients who inject drugs are very high-risk as well.” Despite key informants’ concerns about HIV risk behaviors in local PWID populations, many participants consider themselves to have low HIV risk in part because they tried to avoid sharing syringes. However, for many participants, despite initial responses about avoiding syringe sharing and therefore having low HIV risk, further discussion revealed that many participants actually engaged in high-risk behaviors for HIV transmission with relative frequency. These behaviors included syringe sharing and condomless sex when “in a bind” and needing money, drugs, or housing. As Skyler, a 35-year-old Hispanic genderqueer participant in Providence explained:

I’m careful. I’ve been really careful with my drug use…I’ve done riskier stuff...like the other day, I shared needles, my needle broke. [The NSP] was closed...I didn’t have money, I felt sick…I try to clean [my syringes] as much as possible, but I’ve been sharing cookers, which is something I didn’t used to do…and also doing sex work.

Another individual-level barrier to PrEP use was concern about PrEP side effects and health consequences, including whether HIV medications would work if they became HIV-infected after taking PrEP. Regarding extended use of PrEP, Chris, a 43-year-old White man in Boston participant, asked, “You don’t think your body’s going to find a way to mutate and adapt to be able to go around that?” Participants were also concerned about how PrEP might reduce the efficacy of prescribed medications (e.g., antidepressants), worsen “dope sickness,” or “mess with my methadone,” highlighting unique concerns of PWID that PrEP educational campaigns and interventions will need to address.

Finally, participants and key informants emphasized the significance of competing priorities in PWID lives that could challenge PrEP utilization, including the demands imposed by physical dependence and withdrawal symptoms. For example, many participants explained that “the drugs take precedence over everything else,” and alleviating withdrawal “comes first.” Drug use could pose particular challenges to maintaining daily adherence, as Patrick, a 49-year-old White man in Providence, explained, “Things always come up, drugs, you know…they come first. We’re druggies, you know? So, I’d be forgetful, I’m sure.” Drug use could also interfere with attending scheduled appointments for PrEP screening, initiation, and follow-up care. When asked about keeping health-related appointments, Greg, a 52-year-old White man in Boston, responded, “I blow them off because I get high. I drink in the morning, and I get high in the afternoon, and I don’t want to go to the doctor either way.” Given the longitudinal nature of PrEP, key informants also worried about PWID “disappearing” in between PrEP appointments. One CBO outreach worker in Boston reported:

I may have a guy that’s coming in here [for] three weeks, four weeks…ten weeks, and then he drops off the face of the earth. Now, is he arrested? There’s so much that can go wrong or happen day to day for them.

Although most participants stated that drug-related competing priorities could interfere with PrEP uptake and adherence, a few participants and key informants believed that, with appropriate supports, PWID could be adherent to PrEP. Many participants had experience with daily medications including methadone, and one key informant, a clinical provider in Boston, described heroin use as “a much more routinized way of using drugs,” in which individuals were “basically taking their heroin like medicine.” Similarly, as Kevin, a 42-year-old White man in Boston, rhetorically asked, “I’m so good at taking drugs; why can’t I take something that’s good for me?”

### Interpersonal-level barriers

Interpersonal-level barriers to PrEP utilization included negative experiences interacting with healthcare providers and HIV-related stigma within social networks. First, participants’ past interactions with healthcare providers were overwhelmingly negative, and several described experiencing stigma or mistreatment that made them reluctant to disclose their drug use or other risk behaviors when seeking healthcare. Donna, a 43-year-old White woman in Providence, explained:

The minute [the doctors] find out you are a drug addict, that you are an injection [drug] user, you can see it right in their face. They change their whole attitude. They do not want to help you…I hate telling the doctor that I use drugs…because they are going to blame anything wrong with you on the drug use.

Key informants contextualized patient experiences within healthcare systems that were not adequately equipped to address the complex needs of PWID, as described by a clinical provider in Boston:

We do not get very much education…surrounding addiction…What has helped me the most, in terms of being comfortable and confident providing medical care to people who inject drugs, is understanding more about the patient perspective, what their life is like, how often they inject, what their injection practices are…Honestly, this population has had a lot of bad experiences [and is] often stigmatized and discriminated against in healthcare settings.

On the other hand, relationships with CBO staff were more positive, as Aaron, a 24-year-old White man in Boston, compared NSP staff to healthcare providers: “People feel more comfortable in a place like [the NSP]. I don’t feel embarrassed to talk to people here. There are some things I wouldn’t tell a doctor that I can tell these people because they know where I’m coming from.” Several participants and key informants suggested that CBOs could be ideal PrEP delivery sites because they employed trusted staff and were often frequented “on a daily basis.” Key informants also believed that CBO-based outreach workers could help facilitate access to PrEP and physicians with more experience in addiction medicine and working with PWID.

In addition to difficulties with providers, participants also discussed HIV-related stigma within their social networks, worrying that others would think they were HIV-infected if they were seen using PrEP. Michael, a 48-year-old Black man in Providence, reported: “I mean, you get your prescription, somebody [sees] it and is like, ‘Oh, he’s taking pills because [he has] AIDS’.” Others were concerned that being known to use PrEP would lead to assumptions about their engagement in specific high-risk behaviors.

### Clinical- and structural-level barriers

PWID and key informants also described important barriers to PrEP use at the clinical and broader structural levels. First, clinical challenges included poor infrastructure for PrEP delivery and low provider capacity or willingness to prescribe PrEP to PWID. Related to clinical infrastructure, in referring to a local clinic frequented by PWID for general healthcare needs, one key informant, a CBO program manager in Boston, noted that it was not prepared to provide PrEP-related care because, “the infrastructure doesn’t really exist right now for [PrEP].” Key informants explained that many PrEP providers were located within infectious disease departments where HIV-uninfected PWID were unlikely to present. Within these settings, CBO-based key informants were also concerned that physicians’ often singular focus on PWID patients’ drug use prevented physicians from considering “drug user health” more holistically or more accurately assessing HIV risk and related PrEP need. This program manager explained:

[HIV] is not their main concern when they are looking at an IDU [injection drug user]. They are not going, “Oh, this would be a candidate for PrEP.” They are more thinking, “Okay, what opioid treatment can we get them on?” No one’s really thinking, “Okay, but they also have all these high risk sexual behaviors” that are just as important as their injection behaviors that everyone’s talking about.

Another clinical barrier to PrEP use involved the process of obtaining PrEP, with some participants and key informants considering PrEP screening and retention protocols to be too burdensome for the PWID population. Jessica, a 35-year-old White woman in Boston, said, “If it’s something that…you have to make a bunch of doctors’ appointments and jump through all these hoops, more than likely, no, us addicts, we wouldn’t follow through with it.” Suggestions for streamlining the PrEP screening process were common across interviews. Key informants identified particular ways to facilitate PrEP uptake and delivery through CBOs. For example, CBOs could partner with PrEP prescribers, as described by one clinical provider in Boston:

If everything is done as far as screening labs, [we could say,] “Hey, come back when you’re gonna pick up your next syringes, and we’ll also gonna check in about how you’re doing... with your first week on PrEP.” I think it’s going to take that type of approach… what would support that group in conjunction with the other things they are accessing services for.

Barriers at the broader structural level related to homelessness, criminal justice system involvement, lack of money or identification to fill prescriptions, and transportation difficulties. First, many PWID in our sample, like Stacey, a 36-year-old White woman in Boston, were homeless or unstably housed and described how the instability in their daily routines could interfere with PrEP uptake and retention:

Just being…on the streets, you never know where you are going to be at that moment. You never know if you are going to be dope-sick [or] running to go make money. You just never know where, every second of every day…You cannot plan. It’s just the life that you live.

Also related to homelessness, participants like Aaron, a 24-year-old White man in Boston, reported difficulty safeguarding medications because “your whole bag can get [stolen]…and you could just completely lose a whole prescription.”

Given the illicit nature of drug use, many PWID described criminal justice system involvement, which some participants and key informants discussed as a barrier to PrEP use. For example, PWID expressed concern that PrEP use could be interrupted by incarceration, which disconnects individuals from regular sources of medication, healthcare, and social services. For example, drawing on her experience with HCV treatment, Megan, a 35-year-old White woman in Boston, reported, “I was offered [HCV treatment] and I was gonna be starting it… but then I went to jail, so yeah…”

Although almost all participants were publicly insured, participants explained that lacking proper identification could be a barrier to utilizing public insurance to receive PrEP from pharmacies. Finally, transportation and locations of services could also present challenges to PrEP access, for which some participants and key informants provided practical suggestions for interventions to provide assistance accessing identification cards and vouchers for transportation options.

## Discussion

PWID have elevated risk for HIV acquisition due to injection and sexual risk behaviors [2]. With recent evidence of HIV transmission and outbreaks related to injection drug use [5, 32], particularly in the context of increasing prevalence of opioid use and injection [1, 9, 10], maximizing the accessibility of all available HIV prevention tools for PWID should be a public health priority. The efficacy of antiretroviral PrEP for HIV prevention among PWID has been established in a major clinical trial [13]; however, to date, PrEP uptake in this socially marginalized population has been low [22–26], and acceptability and accessibility remain understudied. Although participants and key informants in our study described numerous, multilevel barriers to PrEP utilization, interest in PrEP in this population was relatively high [30]. Unfortunately, to the best of our knowledge, no PrEP interventions have been developed for or tested among actively injecting PWID (i.e., outside of treatment settings such as methadone clinics) [26–29]. By combining the formative evidence from this study with behavioral theory known to support HIV-related interventions, we provide the following recommendations for developing evidence- and theory-based strategies for addressing the multilevel barriers to PrEP use among PWID.

As detailed below, Social Cognitive Theory (SCT) could prove useful in the development of interventions to support PrEP utilization among PWID. SCT posits that people, their behaviors, and surrounding environments interact to influence how and when behaviors are performed [40]. Interventions based on SCT have been developed to improve antiretroviral therapy (ART) and PrEP adherence in PWID and other high risk and marginalized populations (e.g., sexual minority men) [41, 42]. SCT identifies key determinants of health behaviors including outcome expectations (beliefs about the consequences of behavioral choices), behavioral capability (actual ability to perform desired behaviors), self-efficacy (belief in one’s ability to monitor and control one’s behaviors), observational learning (belief based on observing role models perform desired behaviors), and the environmental (requiring changes to external factors) [40]. As such, based on our findings regarding the socioecological barriers to PrEP use among PWID and the behavioral determinants identified in SCT, we suggest the following strategies for interventions to help improve PrEP use among PWID. Table 3 provides examples of suggested PrEP intervention activities that we suggest to address major barriers and behavioral determinants.

**Table 3:** Social cognitive theory-informed strategies to address socioecological barriers to PrEP use among PWID

Socioecological PrEP Use Barriers	Social Cognitive Theory targets* and relevant suggested PrEP intervention activities
Individual • Perceived risk • Lack of knowledge about PrEP relevance for PWID, access, and side effects • Competing priorities for attending appointments and taking PrEP • Heavy drug use	Outcome expectations • Compare strategies (e.g., pros/cons) to help PWID see the value in PrEP Behavioral capability • Provide simple messaging to increase knowledge of PrEP, access, and adherence • Direct community and street outreach for education and recruitment through partnering with trusted CBOs • Facilitate goal-setting for attending PrEP appointments and PrEP adherence Self-efficacy • Problem-solving strategies to manage PrEP use in the context of heavy drug use, including use of SMS messaging reminders and coupling taking PrEP with other routines (including drug use) • Outreach workers to maintain contact and support adherence Observational learning • Seeding, or diffusion of knowledge via peer networks, support groups • Facilitate groups of peers that can support one another’s PrEP use
Interpersonal • HIV- and PrEP-related stigma from social, sexual, and drug networks • Distrust of doctors and drug use-related stigma from healthcare providers	Outcome expectations • Identify CBO staff and/or PrEP navigator that can provide support related to stigma, mental health, drug use in the context of PrEP Self-efficacy • Problem solve strategies to respond to and cope with stigma Observational learning • Provide role model examples of responding to HIV-related stigma from social, sexual, and drug networks and engaging in effective patient-provider communication Environment • Cultural competency training for healthcare staff
Clinical/structural • Complex PrEP protocol • Decentralized care • Transportation difficulties • Incarceration	Behavioral capability • Case managers/PrEP navigators to help navigate initial appointments Self-efficacy • Discuss strategies for keeping medications safe, including using non-descript bottles and keeping only small amounts of pills with them and locking the others at CBOs that they frequent • Facilitate goal-setting for attending PrEP appointments and PrEP adherence Environment • Provide flexible appointments/drop-in times • PWID and KI suggested that PrEP providers have clinical time at CBOs/NSPs where PWID already frequent • Develop innovative models to provide PrEP during and following criminal justice system involvement

*Social Cognitive Theory (SCT) relies heavily on the concept of reciprocal determinism, which posits that a dynamic interaction between the person, the behavior, and the environment influence how and when a behavior is performed. As such, an intervention to change behavior must consider multiple methods, including targeting knowledge, self-efficacy, skills, and the environment

First, *outcome expectations* [40], or the belief about the likelihood of consequences of behavioral choices, if improved, could help address a number of the individual-level and interpersonal barriers to PrEP utilization identified in our study. For example, low PrEP knowledge and concerns about side effects and drug interactions, which have also been identified as barriers to ART adherence among HIV-infected PWID [43, 44], could be addressed through educational activities that aim to increase factual knowledge of PrEP while also improving HIV risk perception, which has been shown to strongly predict PrEP interest and uptake [30, 45–48]. By using other strategies such as motivational interviewing [49] and value ranking [50], an interventionist could work with participants to more accurately assess their HIV risk and identify the value of PrEP in their own lives.

*Behavioral capability* [40], or the actual ability to perform desired behaviors, and *self-efficacy* [40], the belief in one’s ability to monitor and control one’s own behaviors, emotions, or thoughts, are highly linked. Targeting these behavioral determinants could help address barriers to PrEP use and adherence at the individual level (e.g., heavy drug use), interpersonal level (e.g., stigma), and structural level (e.g., complex PrEP protocols). For example, by working with patients to brainstorm strategies to manage and problem-solve their own barriers to PrEP use, interventionists could help individuals increase their self-efficacy for PrEP adherence (e.g., coupling taking PrEP with other routines or setting SMS reminders) [51]. Additionally, case managers or “PrEP navigators” could be deployed to support patients in making appointments for PrEP screening and related care and effectively communicating with providers about their healthcare needs and challenges [52]. While structural barriers are often viewed as outside of individual patients’ control, counselors, case managers, or navigators could also help patients assess specific barriers such as transportation and unstable housing to then develop strategies to minimize the impact of these factors on PrEP utilization [53].

*Observational learning* [40], or behavior change from observing role models perform desired behavior, could increase knowledge by improved outreach efforts, expanded marketing, and educational activities by peers or other trusted individuals. Notably, participants suggested that outreach by trusted CBO staff could help promote the diffusion of “word of mouth” information about PrEP and its relevance to this population. Similarly, to ameliorate interpersonal barriers to PrEP use and promote social support [54], interventionists could work with PWID to facilitate access to support networks (e.g., through peer groups or CBOs). Moreover, to help overcome stigma, patients and interventionists could use role modeling to help PWID learn and practice effective strategies for communicating with healthcare providers despite negative past experiences or providers’ low willingness to prescribe PrEP to PWID [55].

Lastly, the *environment* [40], the context in which behavior change occurs that dynamically interacts with individuals and their behavior, must be addressed through multilevel interventions. For example, to completely address stigma relating to HIV and drug use, starting cultural competency trainings among healthcare staff and changing from using stigmatizing language (e.g., “substance user,” “addict,” “narcotic dependent patient”) to neutral, accurate, person-first language (e.g., “person who injects drugs,” “patient with a substance use disorder”) could decrease negative, stigmatizing perceptions among healthcare providers [56–58]. Additionally, because many PWID experienced structural barriers to PrEP use (e.g., homelessness, poverty) and desire simplified PrEP access processes, PrEP could also be delivered through more accessible and trusted settings, such as NSPs and other CBOs (e.g., drop-in HIV/HCV testing centers), as suggested by participants and key informants in our study. Indeed, HCV treatment, which also requires testing and screening processes and daily adherence to medication, has been successfully delivered through drug treatment (e.g., methadone) services [59, 60] and could possibly be delivered through other community-based harm reduction venues (e.g., supervised injection facilities or drug consumption rooms in settings where those services are available) [61]. Through these organizations, PrEP prescribers could provide or make referrals to other essential health services. Indeed, PrEP may only be cost-effective when bundled with other essential services for PWID such as medications for the treatment of opioid use disorder [16, 62]. While NSP coverage is expanding nationwide, pharmacies have also emerged as important settings for delivery of harm reduction services to PWID [4, 63]. Importantly, as participants noted, incarceration could prevent or interrupt PrEP utilization among PWID by disconnecting individuals from regular sources of healthcare and social services. Novel intervention strategies are needed to provide PrEP during criminal justice system involvement and following release from incarceration, a period when injection-related risk behaviors for HIV acquisition are known to be high [64–66].

Our qualitative study had several limitations. While we purposively sampled diverse PWID and key informants, our recruitment of PWID was limited to two urban centers in the Northeastern USA. Thus, our findings may not generalize to other geographic regions or non-urban areas where HIV transmission dynamics and access to health and harm reduction services may differ. Future quantitative studies with larger samples of PWID could help confirm the generalizability of the findings presented in this study while also exploring potential differences in interventions needs for specific sub-groups of this population. With our recruitment efforts focused on PWID accessing services at local CBOs, our sample may not represent individuals who are not engaged in NSPs or other CBOs, or individuals in recovery who may experience different patterns of HIV risk and related PrEP need. Nevertheless, through working with CBOs, we successfully recruited high-risk PWID who could potentially benefit from PrEP and identified PrEP delivery and intervention suggestions that could be feasible within these settings.

Future intervention development research would benefit from understanding the experiences of PWID with actual rather than theoretical PrEP experiences, which we were not able to assess given the lack of participants with PrEP knowledge or experience. Additionally, research is needed to assess the feasibility, acceptability, and efficacy of the intervention strategies proposed above. Through using both a theoretical basis in SCT and grounding in the contextual realities of PWID in the Northeastern USA, the intervention strategies identified through this study provide a promising model for improving PrEP utilization in this high-risk and socially marginalized population.
